# Prevalence of Depression among Healthcare Workers during the COVID-19 Outbreak: A Systematic Review and Meta-Analysis

**DOI:** 10.3390/jcm10153406

**Published:** 2021-07-30

**Authors:** Beatriz Olaya, María Pérez-Moreno, Juan Bueno-Notivol, Patricia Gracia-García, Isabel Lasheras, Javier Santabárbara

**Affiliations:** 1 Research, Innovation and Teaching Unit, Parc Sanitari Sant Joan de Déu, Universitat de Barcelona, 08830 Sant Boi de Llobregat, Spain; beatriz.olaya@sjd.es; 2 Centro de Investigación Biomédica en Red de Salud Mental (CIBERSAM), Ministry of Science and Innovation, 28029 Madrid, Spain; jsantabarbara@unizar.es; 3 Hospitalary Pharmacy Service, Hospital Universitario Miguel Servet, Paseo Isabel la Católica, 1-3, 50009 Zaragoza, Spain; marpermor159@gmail.com; 4 Psychiatry Service, Hospital Universitario Miguel Servet, Paseo Isabel la Católica, 1-3, 50009 Zaragoza, Spain; pgraciag@salud.aragon.es; 5 Department of Microbiology, Pediatrics, Radiology and Public Health, Faculty of Medicine, University of Zaragoza, Building A, 50009 Zaragoza, Spain; isabel.lasheras@hotmail.es; 6 Instituto de Investigación Sanitaria de Aragón (IIS Aragón), Avenue San Juan Bosco, 13, 50009 Zaragoza, Spain

**Keywords:** depressive symptoms, COVID-19, nurses, medical doctors, frontline, pooled prevalence

## Abstract

Background: There is evidence of a high psychological toll from the COVID-19 pandemic in healthcare workers. This paper was aimed at conducting a systematic review and meta-analysis of studies reporting levels of depression among healthcare workers during the COVID-19 and estimating the pooled prevalence of depression. Methods: We searched for cross-sectional studies listed on PubMed from 1 December 2019 to 15 September 2020 that reported prevalence of depression in healthcare workers, nurses, medical doctors, and COVID-19 frontline professionals. The pooled proportions of depression were calculated with random effects models. Results: We identified 57 studies from seventeen countries. The pooled prevalence of depression in healthcare workers was 24% (95% CI: 20–28%), 25% for nurses (95% CI: 18–33%), 24% for medical doctors (95% CI: 16–31%), and 43% for frontline professionals (95% CI: 28–59%). Conclusions: The proportion of depression in nurses and medical doctors during the COVID-19 pandemic was similar to that found in the general population as previously reported in other meta-analyses conducted with smaller numbers of studies. Importantly, almost half of the frontline healthcare workers showed increased levels of depression. There is need for a comprehensive, international response to prevent and treat common mental health problems in healthcare workers.

## 1. Introduction

The new coronavirus (SARS-CoV-2) was first identified in a wet market in Wuhan, Hubei province, China, in December 2019 [1]. This virus causes a highly infectious acute respiratory syndrome (COVID-19) that can be associated with serious pneumonia and eventually lead to death. Due to its rapid spread around the world, the World Health Organization [2] declared the COVID-19 as a pandemic in March 2020, and from its identification to this date (12th November 2020), more than 51.9 million people have been confirmed as cases worldwide, with 1.2 million deaths [3]. The enormous impact on people’s physical and mental health, and on economic systems worldwide, is one of the main challenges for society in this century [4].

Healthcare workers (HCW) are a fundamental part of the global response to COVID-19. Because of their close personal exposure to patients with COVID-19, their risk of infection is very high. A recent study reports HCW to be at an 11.6 times higher risk of infection than the general population, although this risk decreases to 3.4 after accounting for the differences in testing frequency between HCW and the general community [5]. Besides this higher risk of infection, several observational studies conducted during the COVID-19 pandemic have shown that health professionals are at a higher risk of developing psychological problems [6]. Growing patient load and working under pressure in resource-deprived settings might increase psychological stress among HCW [7,8]. They are also more exposed to prolonged work shifts, lack of adequate equipment (i.e., protective equipment (PPE)), and fear of infecting themselves or relatives [9,10]. This fear, in turn, may be associated with anxiety, depression, and insomnia [11,12,13].

HCW thus constitute one of the groups most vulnerable to psychological distress, requiring immediate interventions to improve their wellbeing and the healthcare system capacity. Two very recent systematic reviews and meta-analyses on the prevalence of anxiety and depression have been published, reporting the pooled prevalence among HCW. The first one, conducted by Pappa et al. [14] in April 2020, included a total of thirteen cross-sectional studies (all of them conducted in China except one, from Singapore) reporting a pooled prevalence of anxiety of 23.2% and 22.8% for depression. The second [15] was based on seven studies conducted in China and found an increased risk of anxiety and depression in HCW, compared with other professionals (OR = 1.61; 95%CI 1.33 to 1.96 and OR = 1.32; 95%CI 1.09 to 1.60, respectively).

Due to the rapid, evolving nature of this health emergency, an increasing number of other studies from different countries addressing mental health problems among HCW have been published in recent months. Thus, the present study is aimed at updating and extending the previous work of Pappa et al. [14] and da Silva and Neto [15] by conducting a systematic review and meta-analysis of studies published afterwards reporting a global prevalence of anxiety and depression among HCW during the COVID-19 outbreak.

## 2. Materials and Methods

The present study followed the PRISMA guidelines for reporting systematic reviews and meta-analysis [16] (Supplementary Table S1).

### 2.1. Search Strategy

The search strategy (Supplementary Table S2) included all cross-sectional studies informing about the prevalence of depression that were published from 1 December 2019 to 15 September 2020. The search was conducted by two researchers (MPM and JBN) using MEDLINE via PubMed. Briefly, they focused on depression, although an anxiety term was additionally included to examine whether these articles also included relevant information about depression. Depression could be measured either using diagnostic tools (e.g., structured interviews) or standardized scales to assess depressive symptomatology. As our main objective was to calculate the overall prevalence of depression, in case we found a study using scales, we considered the presence of depression reported according to a certain cut-off point for that given scale. Thus, henceforth we use the term “depression” to refer to either a full-blown diagnosis or presence of depression according to a cut-off point.

Search terms also included samples of HCW, nurses, medical doctors, and/or frontline HCW. There was no language restriction. We inspected references from selected articles to detect additional studies. In case of disagreement, a third and fourth reviewer (JS and IL) were consulted to reach a consensus.

### 2.2. Selection Criteria

The following inclusion criteria for studies were used: (1) studies providing cross-sectional data on the proportion of depression during the COVID-19 outbreak; (2) studies conducted with samples of health care workers; (3) studies in which the assessment methods for depression were described; and (4) studies for which the full-text was available. Studies that used other specific samples (e.g., adolescents and patients) and review articles were excluded from the present study.

We extracted the following data using a pre-designed form: country, sample size, prevalence rates of depression, proportion of women, average age, instruments used to assess depression, response rate, and sampling methods.

### 2.3. Assessment of Methodological Quality

Two independent reviewers (JS and JBN) rated the methodological validity of selected articles before their inclusion in the review using the Joanna Briggs Institute (JBI) standardized critical appraisal instrument for prevalence studies [17]. This tool uses nine criteria to evaluate quality with a score ranging from zero (‘No’) to one (‘Yes’).

In case of disagreement between the two reviewers, there was a discussion to resolve it between them or with a third reviewer (PGG).

### 2.4. Statistical Analysis

We used a generic inverse variance method with a random effect model [18]. To check heterogeneity across studies, we calculated the Hedges *Q* statistic (a *p* value < 0.10 indicates statistical significance) and the *I*^2^ statistic and 95% confidence interval [19]. *I*^2^ values between 25% and 50% are considered low, 50%–75% moderate, and 75% or greater high [20]. Different study designs or demographic characteristics may explain the heterogeneity. Thus, we calculated meta-regression and carried out subgroup analyses [21] to find potential sources of heterogeneity [22]. A sensitivity analysis was also made by omitting studies one by one. This allowed us to learn how each individual study influenced the overall result. Publication bias was determined by visually inspecting a funnel plot. Since funnel plots might inaccurately assess publication bias in meta-analyses of proportion studies [23], we additionally calculated Egger’s [24] and Begg’s tests [25], with *p* values < 0.05 indicating publication bias.

Despite the fact that one inclusion criterion for a given study was the use of HCW samples, we were interested in separately calculating the pooled prevalence for the following groups: HCW in general (with no distinction of the type of worker or working in the frontline), nurses, medical doctors, and frontline HCW. Frontline HCW were those who provided direct care to patients with a diagnosis of infection by COVID-19 or who worked in units where care was provided. Additionally, for practical purposes, the pediatric HCW, physical therapists, and laboratory HCW were considered as HCW. Statistical analyses were conducted with STATA statistical software (version 10.0; College Station, TX, USA) and R [26].

## 3. Results

**Figure 1:**
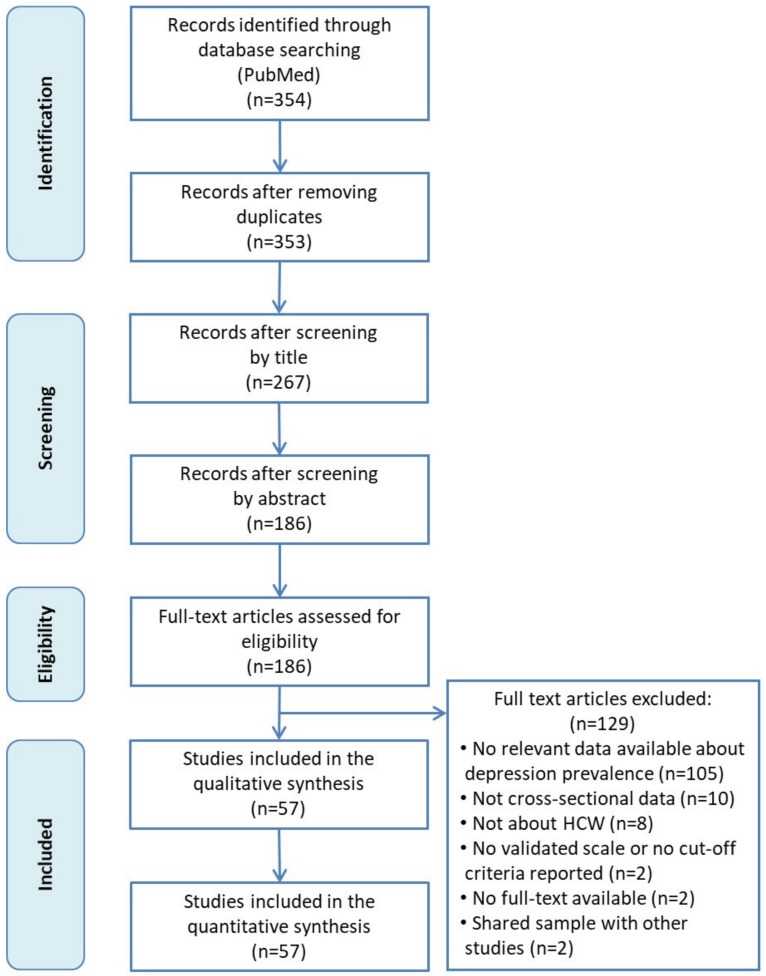
Flowchart of the study selection.

Flowchart of the search strategy and study selection process is shown in Figure 1. We initially identified 354 studies. After removing duplicates and studies after the first screening, 186 articles were read in full. Finally, a total of 57 studies were included in the present meta-analysis [6,12,13,27,28,29,30,31,32,33,34,35,36,37,38,39,40,41,42,43,44,45,46,47,48,49,50,51,52,53,54,55,56,57,58,59,60,61,62,63,64,65,66,67,68,69,70,71,72,73,74,75,76,77,78,79,80].

The results are organized as follows: Table 1 shows the characteristics of those studies (46) that reported prevalence rates of depression in HCW (without distinction in the type of workers); Table 2 displays characteristics of studies reporting data from nurses (14); Table 3 characteristics for medical doctors (10); and Table 4 for frontline HCW (12).

Approximately half of the studies were conducted in China (*n* = 24), but we also found studies from India (*n* = 4), Italy (*n* = 3), Turkey (*n* = 3), Singapore (*n* = 2), and one study from each of the following countries: Cameroon, Croatia, Jordan, Kosovo, Libya, Nepal, Poland, Serbia, South Korea, Spain, Switzerland, and the USA. The sample size ranged from 46 to 14,825 participants, and the mean age ranged from 29 to 47 years. All studies except one included both men and women, with women predominating in most of the studies that reported this (40 out of 43). All studies used online questionnaires and all except two used non-random methods. Twenty-three studies reported response rate, ranging from 20.4% to 94.9%. All studies measured depression using standardized scales, most commonly the Patient Health Questionnaire-9 (PHQ-9, *n* = 21 studies), the Depression, Anxiety, and Stress Scale (DASS-21, *n* = 8 studies), and the Hospital Anxiety and Depression Scale (HADS, *n* = 8 studies).

The risk of bias ranged from 5 to 8, with a mean score of 6.95 (Supplementary Table S3, Table 1). The most common limitations were (a) recruitment of participants not appropriate (56 studies), (b) response rate not reported or large number of non-responders (33 studies), and (c) sample size too small to ensure good precision of the final estimate (19 studies).

**Figure 2:**
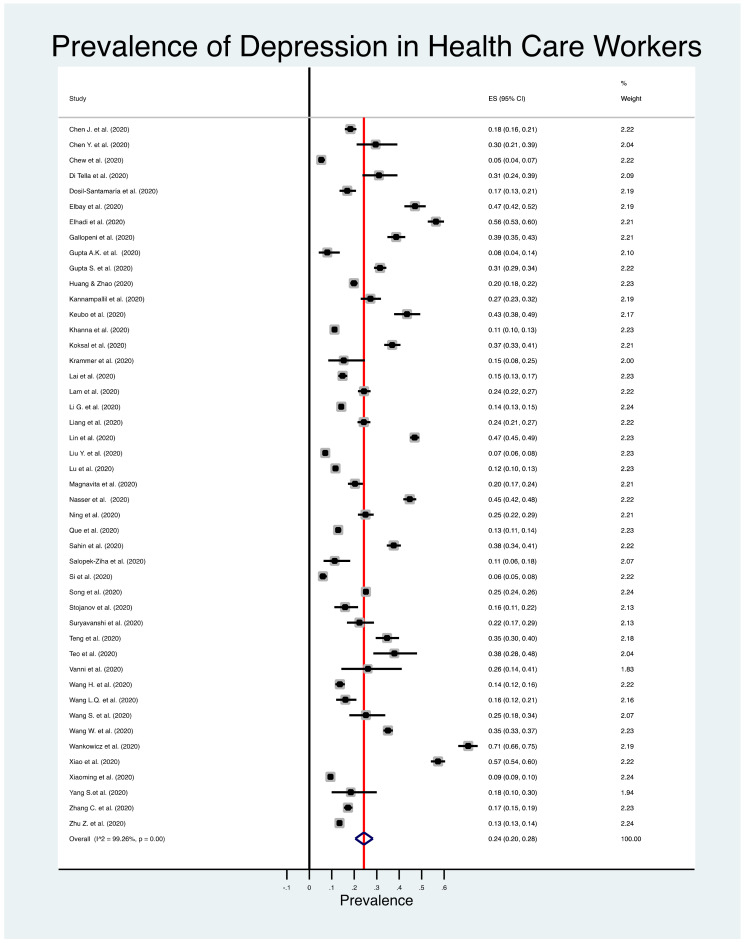
Forest plot for the prevalence of depression among healthcare workers.

**Figure 3:**
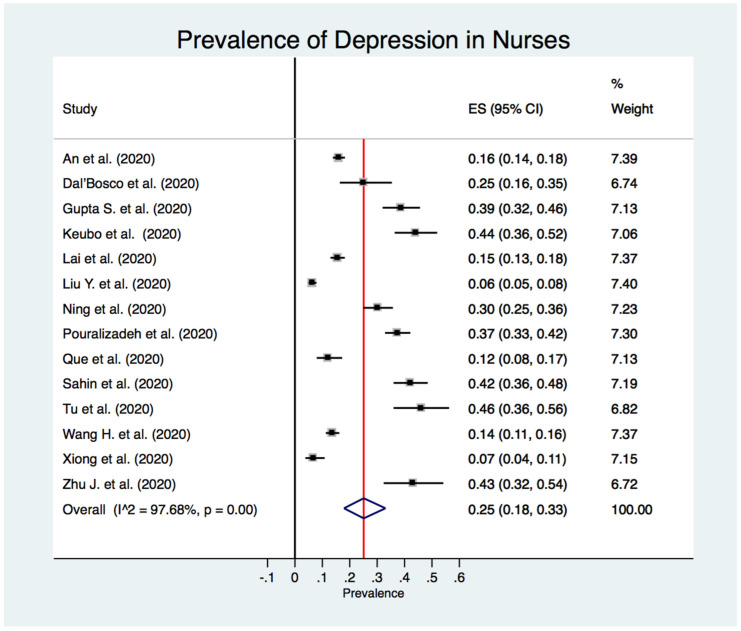
Forest plot for the prevalence of depression among nurses.

**Figure 4:**
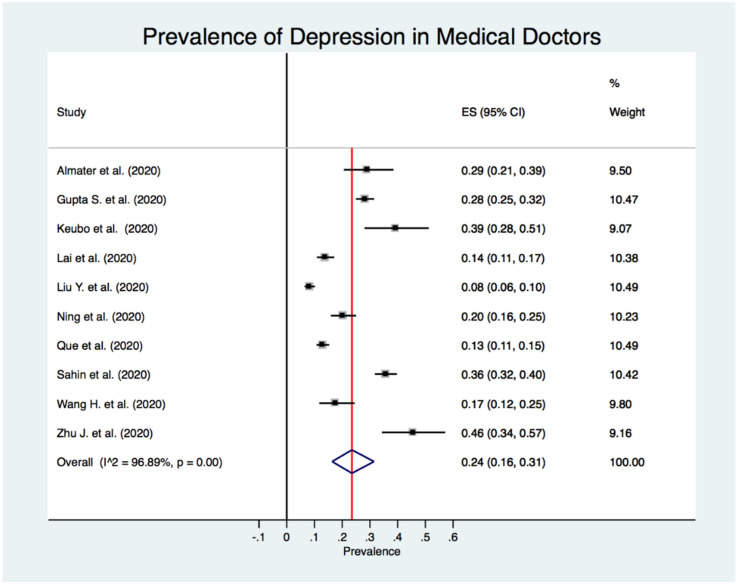
Forest plot for the prevalence of depression among medical doctors.

**Figure 5:**
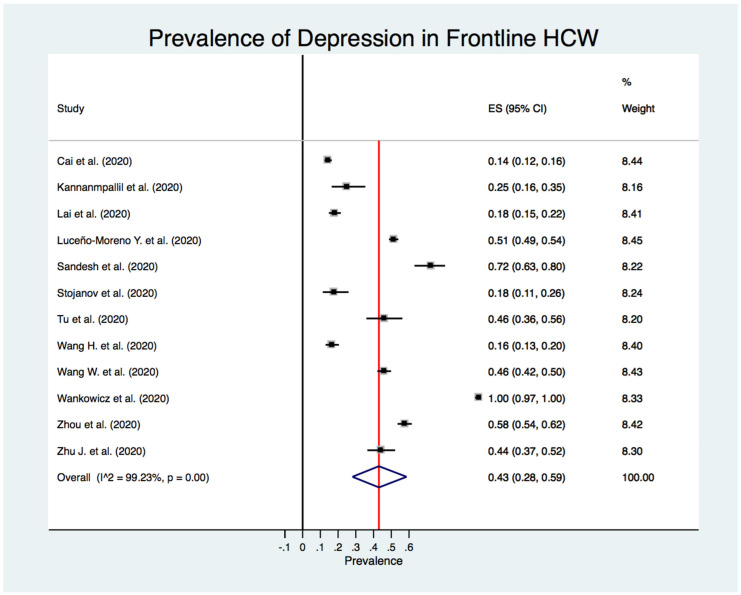
Forest plot for the prevalence of depression among frontline healthcare workers.

Figure 2 shows the estimated overall prevalence of depression in HCW (24%; 95% CI: 20%–28%), 25% in nurses (95% CI: 18%–33%) (Figure 3), 24% in medical doctors (95% CI: 16%–31%) (Figure 4), and 43% in frontline HCW (95% CI: 28%–59%) (Figure 5), with significant heterogeneity between studies (Q test: *p* < 0.001) across these four categories. Additionally, the prevalence of depression in frontline HCW was significantly higher than in HCW overall (*p* < 0.05).

Potential sources of heterogeneity were investigated across the studies. Our subgroup analysis showed that prevalence of depression was lower in studies using the DASS-21, those carried out in China, studies using convenience sampling methods and those of high methodological quality (Table 5).

The exclusion of studies one-by-one from the analysis did not substantially change the overall prevalence rate of depression. Thus, no single study had a disproportional impact on the overall prevalence (data not shown).

**Figure 6:**
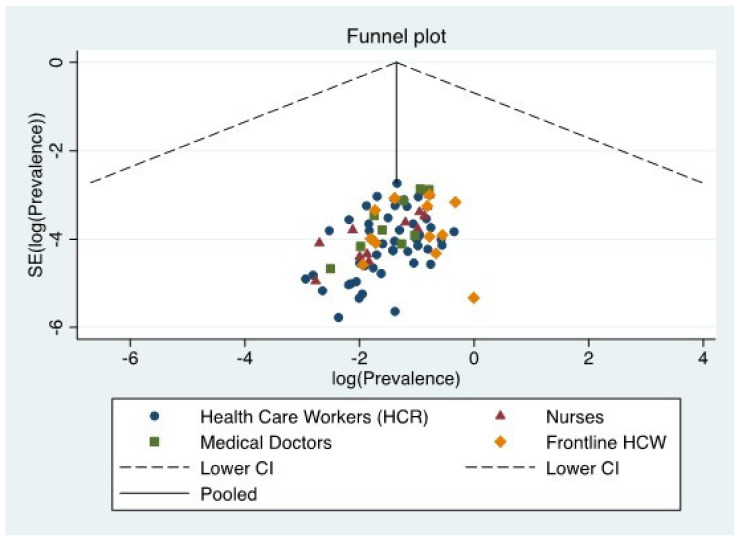
Funnel plot for the prevalence of depression.

Visual inspection of the funnel plot (Figure 6) suggested a small publication bias for the prevalence estimate in HWC, nurses, and medical doctors, confirmed by significant results in the Egger’s test (*p* < 0.05). However, no publication bias was detected for frontline HCW (Egger’s test: *p* = 0.928).

## 4. Discussion

The present systematic review and meta-analysis identified a total of 57 cross-sectional studies reporting rates of depression among HCW. The pooled prevalence rate of depression in HCW was 24%, and when analyzing professional groups, we found that the rates were similar in nurses (25%) and medical doctors (24%), whereas up to 43% of frontline HCW report depression. The overall prevalence of depression found in HCW, nurses and medical doctors is similar to that found in a recent meta-analysis conducted from January 2020 to May 2020. This meta-analysis was based on 12 population-based studies conducted during the COVID-19 outbreak, finding that the overall prevalence was 25% in the general population [81].

Since the outbreak of COVID-19 pandemic in January 2020, the attention paid to the impact on mental health among HCW has grown exponentially, as indicated by the large number of studies found. A recent systematic review and meta-analysis of the prevalence of depression, anxiety, and insomnia among HCW during the pandemic included thirteen studies published up to 17 April 2020. In that study, the pooled prevalence of depression was 22.8%, based on ten studies [14]. A subgroup analysis for different occupational categories found that the pooled prevalence for nurses was 30.3% and for medical doctors 25.9%. These figures are slightly higher than those reported in our meta-analysis. These discrepancies might be explained by the different number of studies included. In the Pappa et al. meta-analysis, only five studies were considered in calculating separate pooled prevalence of depression for nurses and doctors, whereas our study was based on 14 and 10 studies to calculate prevalence depression in nurses and doctors, respectively. Another remarkable difference between the two meta-analyses is the origin of the samples, with the Pappa et al. study mainly focused on a Chinese population. However, our meta-analysis includes a broad range of countries from very different regions worldwide. This regional heterogeneity, along with a greater sample size, allows us to provide an updated estimation of the pooled prevalence of depression among HCW.

Our pooled prevalence of depression found in HCW (25%) is also higher compared with another systematic review and meta-analysis based on samples of HCW that reported a prevalence of 12.2% [15]. The study was conducted April–May 2020 and was based on seven cross-sectional studies, all of them conducted in China. Again, the diversity of the origin of the samples and/or the number of studies included might explain these discrepancies.

Note that all the studies included in our meta-analysis used self-reported standardized questionnaires to assess depressive symptomatology. Additionally, the use of a great variety of scales might have led to differences in the estimation of the presence of depression. In fact, our results show that those studies using the DASS-21 questionnaire reported lower prevalence rates of depression. Despite the convenience of using the same instruments and the inclusion of a diagnosis based on clinical interviews, this is not always possible in epidemiological studies.

Similarly, Muller et al. [82] conducted a rapid systematic review in May 2020 focusing on several outcomes such as mental health problems and risk or resilience factors from quantitative and qualitative data. They found a total of 19 studies, with a percentage of depression ranging from 5% to 51%, and a median of 21%. According to their systematic review, the most common risk factors for mental health problems in HCW were being a woman, being exposed to infected patients, and the worry of being infected.

An important contribution of the present meta-analysis is the calculation of the pooled prevalence of depression in frontline HCW. The prevalence is significantly higher (43%) compared with other types of HCW. The mental toll of working at the frontline during previous pandemic outbreaks, such as that of severe acute respiratory syndrome (SARS) and Middle East respiratory syndrome (MERS), has previously been reported to be high [83,84]. The fear of being infected, stigmatization and uncertainty put these workers under extraordinary stress. A qualitative study conducted among frontline HCW in Wuhan (China) pointed up the intensive work (i.e., long working hours and use of personal protective equipment), fear of infecting others or being infected, managing relationships under stressful situations, and feeling powerless to handle patients’ conditions as common experiences during the COVID-19 outbreak [85].

However, factors contributing to increased vulnerability to depression among HCW as well as resilience characteristics (such as coping strategies) might be culturally different. Additionally, inequalities related to health systems and resources across high- and low-income countries might also contribute to the differing impact of COVID-19 on mental health among HCW from diverse settings. Furthermore, note that the first wave of COVID-19 was characterized by a high degree of uncertainty about the illness, its treatment, and its prognosis. Since the outbreak, there has been an intensive international response to fight the virus along with a research agenda aimed at finding effective treatments for infected patients and preventing the spread of the virus (e.g., vaccines). This means that COVID-19 is a rapid, evolving health challenge that requires up-to-date data to ensure appropriate surveillance of mental health, and specifically for vulnerable subpopulations such as HCW.

There are limitations to be considered when interpreting our results. First, the majority of studies included in the present meta-analysis used convenience samples, so representativeness of HCW might be jeopardized. Second, depression was mainly assessed by means of self-reported data drawn from questionnaires, which might have introduced biases such as social desirability [86], as well as being less accurate than clinical interviews. Third, the inclusion of cross-sectional studies makes it difficult to determine causal associations between the pandemic and depression. Fourth, some of the studies included in the calculation of the pooled prevalence for the three groups of HCW (i.e., nurses, medical doctors, and frontline HCW) might have also been included in the calculation of the pooled prevalence for HCW. Thus, caution should be taken when interpreting our results. Fifth, our systematic review was only conducted in a medical database (MEDLINE); thus, some articles, especially those related to psychology, might not be included. Finally, we found some sources of heterogeneity. For example, using convenience sampling methods, conducting studies in China, and using the DASS-21 questionnaire were associated with lower prevalence rates of depression. Related to this, half of the studies were carried out with Chinese samples, so the results of the present meta-analysis should be approached with caution. Future studies should endeavor to investigate the prevalence of depression among HCW in other countries, and use randomized sampling designs whenever possible, as well as longitudinal designs to determine the evolution of mental health problems in this population.

In summary, our meta-analysis shows that depression during the COVID-19 pandemic is a common mental condition among HCW, with the frontline HCW especially affected. A unified response to help HCW during the pandemic should be placed in the international agenda. Comprehensive psychological support, along with regular and intensive training for HCW, can help safeguard their well-being [87]. Common mental problems, such as depression, should be routinely assessed to detect those HCW at high risk of mental disorders and in need of intensive interventions to alleviate their symptomatology.

## Figures and Tables

**Table 1:** Characteristics of the studies included in the meta-analysis based on samples of healthcare workers.

Author (Publication Year)	Population	Country	Mean Age (SD)	% Females (n)	Sample Size (n)	Response Rate (%)	Sampling Method	Depression Assessment	Time Frame Assessment	Diagnostic Criteria	Prevalence		Quality Assessment *
											%	n	
Chen J. et al. (2020)	HCW	China	36.54 (8.57)	68.63% (619)	902	NR	Convenience sampling	PHQ-9	Last 2 weeks	≥10	18.29%	165	7
Chen Y. et al. (2020)	Pediatric HCW	China	32.6 (6.5)	90.48% (95)	105	84.68%	NR	SDS	Past several days	≥53	29.52%	31	7
Chew et al. (2020)	HCW	India, Singapore	29 (NR)	64.3% (583)	906	90.60%	NR	DASS-21	Last week	≥14	5.30%	48	8
Di Tella et al. (2020)	HCW	Italy	42.9 (11.2)	72.4% (105)	145	NR	Convenience sampling	BDI-II	Last 2 weeks	>13	31.03%	45	6
Dosil Santamaría et al. (2020)	HCW	Spain	42.8 (10.2)	80.29% (338)	421	NR	Snowball sampling	DASS-21	Last week	≥14	16.86%	71	7
Elbay et al.(2020)	HCW	Turkey	36.05 (8.69)	56.8% (251)	442	NR	Convenience sampling	DASS-21	Last week	≥14	47.06%	208	7
Elhadi et al. (2020)	HCW	Libya	33.3 (7.4)	51.94% (387)	745	93.13%	Convenience sampling	HADS	Last week	>10	56.38%	420	8
Gallopeni et al. (2020)	HCW	Kosovo	39 (10.37)	61.32% (363)	592	NR	NR	HADS	Last week	>10	38.68%	229	7
Gupta A.K. et al. (2020)	HCW	Nepal	29.5 (6.1)	52.67% (79)	150	NR	Snowball sampling	PHQ-9	Last 2 weeks	≥10	8.00%	12	6
Gupta S. et al. (2020)	HCW	India	NR	36.12% (406)	1124	79.45%	Quota sampling	HADS	Last week	>7	31.49%	354	8
Huang & Zhao (2020)	HCW	China	NR	NR	2250	85.30%	Convenience sampling	CES-D	Last 2 weeks	>28	19.82%	446	7
Kannampallil et al. (2020)	HCW	USA	NR	54.96% (216)	393	28.58%	NR	DASS-21	Last week	≥10	27.23%	107	6
Keubo et al. (2020)	HCW	Cameroon	NR	54.45% (159)	292	NR	Snowball sampling	HADS	Last week	>10	43.49%	127	6
Khanna et al. (2020)	HCW	India	42.5 (12.05)	43.44% (1023)	2355	NR	NR	PHQ-9	Last 2 weeks	≥10	11.21%	264	7
Koksal et al. (2020)	HCW	Turkey	35.6 (8.5)	70.1% (492)	702	NR	NR	HADS	Last week	>7	36.89%	259	7
Krammer et al. (2020)	HCW	Switzerland	42.6 (13.5)	74.00% (74)	85	76.92%	Convenience sampling	PHQ-9	Last 2 weeks	≥10	15.29%	13	7
Lai et al. (2020)	HCW	China	NR	76.69% (964)	1257	68.69%	Convenience sampling	PHQ-9	Last 2 weeks	≥10	14.80%	186	8
Lam et al. (2020)	HCW	China	NR	75.21% (701)	932	59.51%	Convenience sampling	PHQ-9	Last 2 weeks	≥9	24.36%	227	7
Li et al. (2020)	HCW	China	NR	100% (4369)	4369	82.17%	Convenience sampling	PHQ-9	Last 2 weeks	≥10	14.21%	621	8
Liang et al. (2020)	HCW	China	NR	81.31% (731)	899	NR	Convenience sampling	PHQ-9	Last 2 weeks	≥10	24.25%	218	7
Lin et al. (2020)	HCW	China	NR	NR	2316	NR	Convenience sampling	PHQ-9	Last 2 weeks	>5	46.89%	1086	6
Liu et al. (2020)	Pediatric HCW	China	NR	85.52% (1737)	2031	NR	Convenience sampling	DASS-21	Last week	≥14	7.09%	144	7
Lu et al. (2020)	HCW	China	NR	77.64% (1785)	2299	94.88%	NR	HAMD	Last week	≥7	11.66%	268	8
Magnavita et al. (2020)	HCW	Italy	NR	70.10% (417)	595	73.46%	Convenience sampling	GADS	Last 2 weeks	≥2	20.34%	121	8
Naser et al. (2020)	HCW	Jordan	NR	56,1% (653)	1163	NR	Convenience sampling	PHQ-9	Last 2 weeks	≥10	44.71%	520	7
Ning et al. (2020)	HCW	China	NR	72.88% (446)	612	NR	Snowball sampling	SDS	Past several days	≥53	25.00%	153	7
Que et al. (2020)	HCW	China	31.06 (6.99)	69.06% (1578)	2285	NR	Convenience sampling	PHQ-9	Last 2 weeks	≥10	12.82%	293	7
Sahin et al. (2020)	HCW	Turkey	NR	66.03% (620)	939	NR	Convenience sampling	PHQ-9	Last 2 weeks	≥10	37.59%	353	7
Salopek-Žiha et al. (2020)	HCW	Croatia	NR	NR	124	NR	Convenience sampling	DASS-21	Last week	≥14	11.29%	14	5
Si et al. (2020)	HCW	China	NR	70.68% (610)	863	76.00%	Convenience sampling	DASS-21	Last week	≥14	6.03%	52	8
Song et al. (2020)	HCW	China	34 (8.2)	64.3% (NR)		NR	Convenience sampling	CES-D	Last 2 weeks	≥16	25.18%	3733	7
Stojanov et al. (2020)	HCW	Serbia	40.5 (8.37)	66.17% (133)	201	NR	NR	SDS	Past several days	≥60	15.92%	32	6
Suryavanshi et al. (2020)	HCW	India	NR	51.27% (101)	197	20.40%	Snowball sampling	PHQ-9	Last 2 weeks	≥10	22.34%	44	6
Teng et al. (2020)	HCW	China	NR	NR	338	NR	Snowball sampling	PHQ-9	Last 2 weeks	≥5	34.62%	117	6
Teo et al. (2020)	Laboratory HCW	Singapore	34 (NR)	73.77% (90/122)	103	84.43%	NR	SDS	Past several days	≥60	37.86%	39	7
Vanni et al. (2020)	HCW	Italy	47 (10.37)	65.22% (30)	46	90.20%	Convenience sampling	DASS-21	Last week	≥14	26.09%	12	7
Wang H. et al. (2020)	HCW	China	NR	85.84% (897)	1045	73.18%	Convenience sampling	HADS	Last week	>10	13.59%	142	7
Wang L.Q. et al. (2020)	HCW	China	37 (NR)	77.37% (212)	274	NR	Convenience sampling	PHQ-9	Last 2 weeks	≥10	16.06%	44	6
Wang S. et al. (2020)	Pediatric HCW	China	33.75 (8.41)	90.24% (111)	123	52.44%	Convenience sampling	SDS	Past several days	≥50	25.20%	31	7
Wang W. et al. (2020)	HCW	China	33.5 (8.89)	64.52% (1291)	2001	72.06%	Convenience sampling	HADS	Last week	>7	35.03%	701	8
Wankowicz et al. (2020)	HCW	Poland	40.25 (5.25)	52.15% (230)	441	NR	NR	PHQ-9	Last 2 weeks	>5	70.75%	312	7
Xiao et al. (2020)	HCW	China	NR	67.22% (644)	958	NR	Convenience sampling	HADS	Last week	>7	57.31%	549	6
Xiaoming et al. (2020)	HCW	China	33.25 (8.26)	77.93% (6874)	8817	90.62%	Convenience sampling	PHQ-9	Last 2 weeks	≥10	9.41%	830	8
Yang et al. (2020)	Physical therapists	South Korea	NR	47.69% (31)	65	89.04%	Convenience sampling	PHQ-9	Last 2 weeks	≥10	18.46%	12	7
Zhang et al. (2020)	HCW	China	NR	82.73% (1293)	1563	80.32%	Convenience sampling	PHQ-9	Last 2 weeks	≥10	17.21%	269	8
Zhu et al. (2020)	HCW	China	NR	85.03% (4304)	5062	77.07%	Convenience sampling	PHQ-9	Last 2 weeks	≥10	13.45%	681	8

Note. * Quality score based on the Joanna Briggs Institute (JBI) standardized critical appraisal instrument for prevalence studies [17] (see Supplementary Table S3). NR = not reported; BDI-II = Beck depression inventory-second edition; CES-D = Center for Epidemiologic Studies-Depression scale; DASS-21 = Depression, Anxiety and Stress scales; GADS = Goldberg Anxiety and Depression Scale; HADS = Hospital Anxiety and Depression Scale; HAMD = Hamilton Depression Rating Scale; PHQ-9 = Patient Health Questionnaire; SDS = Zung’s Self-Rating Depression Scale.

**Table 2:** Characteristics of the studies included in the meta-analysis based on samples of nurses.

Author (Publication Year)	Population	Country	Mean Age (SD)	% Females (n)	Sample Size (n)	Response Rate (%)	Sampling Method	Depression Assessment	Time Frame Assessment	Diagnostic Criteria	Prevalence		Quality Assessment *
											%	n	
An et al. (2020)	Nurses	China	32.2 (7.61)	90.75% (1001)	1103	NR	Snowball sampling	PHQ-9	Last 2 weeks	≥10	15.96%	176	7
Dal’Bosco et al. (2020)	Nurses	Brazil	NR	79% (89.8)	88	18.49%	Convenience sampling	HADS	Last week	>7	25.00%	22	6
Gupta S. et al. (2020)	Nurses	India	NR	NR	207	79.45%	Quota sampling	HADS	Last week	>7	38.65%	80	6
Keubo et al. (2020)	Nurses	Cameroon	NR	NR	168	NR	Snowball sampling	HADS	Last week	>10	44.05%	74	5
Lai et al. (2020)	Nurses	China	NR	90.84% (694)	764	68.69%	Convenience sampling	PHQ-9	Last 2 weeks	≥10	15.45%	118	8
Liu Y. et al. (2020)	Nurses	China	NR	NR	1173	NR	Convenience sampling	DASS-21	Last week	≥14	6.31%	74	6
Ning et al. (2020)	Nurses	China	NR	97.97% (289)	295	NR	Snowball sampling	SDS	Past several days	≥53	30.17%	89	6
Pouralizadeh et al. (2020)	Nurses	Iran	36.34 (8.74)	95.2% (420)	441	NR	NR	PHQ-9	Last 2 weeks	≥10	37.41%	165	7
Que et al. (2020)	Nurses	China	35.94 (8.17)	97.75% (195)	208	NR	Convenience sampling	PHQ-9	Last 2 weeks	≥10	12.02%	25	6
Sahin et al. (2020)	Nurses	Turkey	NR	NR	254	NR	Convenience sampling	PHQ-9	Last 2 weeks	≥10	42.13%	107	5
Tu et al. (2020)	Frontline Nurses	China	34.44 (5.85)	100% (100)	100	100%	Cluster Sampling	PHQ-9	Last 2 weeks	≥5	46.00%	46	8
Wang H. et al. (2020)	Nurses	China	NR	NR	773	73.18%	Convenience sampling	HADS	Last week	>10	13.58%	105	7
Xiong et al. (2020)	Nurses	China	NR	97.31 (217)	223	61.80%	Convenience sampling	PHQ-9	Last 2 weeks	≥10	6.73%	15	7
Zhu J. et al. (2020)	Frontline Nurses	China	NR	NR	86	NR	Convenience sampling	SDS	Past several days	≥50	43.02%	37	5

Note. * Quality score based on the Joanna Briggs Institute (JBI) standardized critical appraisal instrument for prevalence studies [17] (see Supplementary Table S3). NR = not reported; DASS-21 = Depression, Anxiety and Stress scales; HADS = Hospital Anxiety and Depression Scale; PHQ-9 = Patient Health Questionnaire; SDS = Zung’s Self-Rating Depression Scale.

**Table 3:** Characteristics of the studies included in the meta-analysis based on samples of medical doctors.

Author (Publication Year)	Population	Country	Mean Age (SD)	% Females (n)	Sample Size (n)	Response Rate (%)	Sampling Method	Depression Assessment	Time Frame Assessment	Diagnostic Criteria	Prevalence		Quality Assessment *
											%	n	
Almater et al. (2020)	MD	Saudi Arabia	32.9 (9.6)	43.9% (47)	107	30.60%	Convenience sampling	PHQ-9	Last 2 weeks	≥10	28.97%	31	6
Gupta S. et al. (2020)	MD	India	NR	NR	749	79.45%	Quota sampling	HADS	Last week	>7	28.17%	211	7
Keubo et al. (2020)	MD	Cameroon	NR	NR	74	NR	Snowball sampling	HADS	Last week	>10	39.19%	29	5
Lai et al. (2020)	MD	China	NR	54.77% (270)	493	68.69%	Convenience sampling	PHQ-9	Last 2 weeks	≥10	13.79%	68	8
Liu Y. et al. (2020)	MD	China	NR	NR	858	NR	Convenience sampling	DASS-21	Last week	≥14	8.16%	70	6
Ning et al. (2020)	MD	China	NR	49.53% (157)	317	NR	Snowball sampling	SDS	Past several days	≥53	20.19%	64	6
Que et al. (2020)	MD	China	33.69 (7.44)	63.49% (546)	860	NR	Convenience sampling	PHQ-9	Last 2 weeks	≥10	12.91%	111	7
Sahin et al. (2020)	MD	Turkey	NR	NR	580	NR	Convenience sampling	PHQ-9	Last 2 weeks	≥10	35.69%	207	6
Wang H. et al. (2020)	MD	China	NR	NR	149	73.18%	Convenience sampling	HADS	Last week	>10	17.45%	26	6
Zhu J. et al. (2020)	Frontline MD	China	NR	64.56% (51)	79	NR	Convenience sampling	SDS	Past several days	≥50	45.57%	36	6

Note. * Quality score based on the Joanna Briggs Institute (JBI) standardized critical appraisal instrument for prevalence studies [17] (see Supplementary Table S3). NR = not reported; DASS-21 = Depression, Anxiety and Stress scales; HADS = Hospital Anxiety and Depression Scale; PHQ-9 = Patient Health Questionnaire; SDS = Zung’s Self-Rating Depression Scale.

**Table 4:** Characteristics of the studies included in the meta-analysis based on samples of frontline healthcare workers.

Author (Publication Year)	Population	Country	Mean Age (SD)	% Females (n)	Sample Size (n)	Response Rate (%)	Sampling Method	Depression Assessment	Time Frame Assessment	Diagnostic Criteria	Prevalence		Quality Assessment *
											%	n	
Cai et al. (2020)	Frontline HCW	China	30.6 (8.8)	68.82% (819)	1173	NR	Non-probabilistic sampling	PHQ-9	Last 2 weeks	≥10	14.32%	168	7
Kannampallil et al. (2020)	Frontline HCW	USA	NR	51.38% (112)	218	15.85%	NR	DASS-21	Last week	≥10	27.98%	61	5
Lai et al. (2020)	Frontline HCW	China	NR	NR	522	68.69%	Convenience sampling	PHQ-9	Last 2 weeks	≥10	18.01%	94	7
Luceño-Moreno et al. (2020)	Frontline HCW	Spain	43.88 (10.82)	86.40% (1228)	1422	92.40%	Non probabilistic sampling	HADS	Last week	≥7	51.34%	730	8
Sandesh et al. (2020)	Frontline HCW	Pakistan	NR	42.86% (48)	112	NR	Convenience sampling	DASS-21	Last week	≥14	72.32%	81	5
Stojanov et al. (2020)	Frontline HCW	Serbia	39.1 (7.3)	65.25% (77)	118	NR	NR	SDS	Past several days	≥60	17.80%	21	6
Tu et al. (2020)	Frontline Nurses	China	34.44 (5.85)	100% (100)	100	100%	Cluster Sampling	PHQ-9	Last 2 weeks	≥5	46.00%	46	8
Wang H. et al. (2020)	Frontline HCW	China	NR	NR	401	73.18%	Convenience sampling	HADS	Last week	>10	16.46%	66	7
Wang W. et al. (2020)	Frontline HCW	China	NR	59.46% (393)	661	72.06%	Convenience sampling	HADS	Last week	>7	45.99%	304	8
Wankowicz et al. (2020)	Frontline HCW	Poland	40.47 (4.93)	56.31% (116)	206	NR	NR	PHQ-9	Last 2 weeks	>5	99.51%	205	6
Zhou et al. (2020)	Frontline HCW	China	35.77 (8.13)	81.19% (492)	606	NR	NR	PHQ-9	Last 2 weeks	>5	57.59%	349	7
Zhu J. et al. (2020)	Frontline HCW	China	34.16 (8.06)	83.03% (137)	165	NR	Convenience sampling	SDS	Past several days	≥50	44.24%	73	6

Note. * Quality score based on the Joanna Briggs Institute (JBI) standardized critical appraisal instrument for prevalence studies [17] (see Supplementary Table S3). NR = not reported; DASS-21 = Depression, Anxiety and Stress scales; HADS = Hospital Anxiety and Depression Scale; PHQ-9 = Patient Health Questionnaire; SDS = Zung’s Self-Rating Depression Scale.

**Table 5:** Overall prevalence rates of depression according to study characteristics.

	Healthcare Workers			Nurses			Medical Doctors			Frontline Healthcare Workers		
	No. Studies	Prevalence (%) (95% CI)	*p* *	No. Studies	Prevalence (%) (95% CI)	*p* *	No. Studies	Prevalence (%) (95% CI)	*p* *	No. Studies	Prevalence (%) (95% CI)	*p* *
Depression assessment			0.531			0.636			0.964			0.600
PHQ-9	20	23 (17–29)		7	23 (14–34)		4	22 (11–35)		5	50 (18–81)	
HADS	43	43 (35–51)		4	29 (14–48)		3	27 (18–38)		2	51 (44–58)	
DASS-21	9	16 (9–24)		1	6 (5–8)		1	8 (6–10)		3	37 (19–57)	
SDS	5	26 (20–32)		2	33 (28–38)		2	25 (21–29)		2	32 (27–38)	
CES-D	2	24 (24–25)		-	-		-	-		-	-	
Other (BDI-II/HAMD/GADS)	3	20 (11–31)		-	-		-	-		-	-	
Country			0.087			**0.031**			0.067			0.235
China	23	21 (16–25)		8	21 (15–27)		6	18 (12–24)		7	33 (19–49)	
Other	23	28 (21–36)		6	38 (33–43)		4	32 (27–38)		5	57 (25–86)	
Sampling method			0.803			0.058			0.508			0.900
Convenience	29	23 (19–28)		8	19 (11–27)		7	22 (13–32)		5	38 (21–57)	
Other	7	25 (18–33)		5	34 (21–48)		3	28 (20–36)		3	36 (10–63)	
Quality rating			0.440			0.356			0.314			0.307
Medium (< 7)	11	28 (19–37)		8	29 (15–44)		7	27 (15–40)		5	55 (18–90)	
High (≥ 7)	35	23 (19–27)		6	21 (13–30)		3	18 (12–24)		7	34 (20–50)	

* *p* value obtained from univariate meta-regression. In bold, significant associations.

## Data Availability

Data sharing is not applicable to this article as no new data were created or analyzed in it.

## Supplementary Materials

The following are available online at https://www.mdpi.com/article/10.3390/jcm10153406/s1, Table S1: PRISMA checklist, Table S2: Search strategy in MEDLINE via Pubmed, Table S3: Quality assessment with the JBI Appraisal Checklist for Prevalence Studies.
