# Supplementary material for: Prevalence of Depression among Healthcare Workers during the COVID-19 Outbreak: A Systematic Review and Meta-Analysis
Source: J Clin Med. 2021 Jul 30;10(15):3406. doi: 10.3390/jcm10153406 (PMC8348388; doi:10.3390/jcm10153406)
Supplement: Supplementary file 1 [file jcm-10-03406-s001.zip › jcm-1305073-supplementary.pdf]

Table S1. PRISMA Checklist.

Section/topic	#	Checklist item	Reported on page #
TITLE			
Title	1	Identify the report as a systematic review, meta-analysis, or both.	1
ABSTRACT			
Structured summary	2	Provide a structured summary including, as applicable: background; objectives; data sources; study eligibility criteria, participants, and interventions; study appraisal and synthesis methods; results; limitations; conclusions and implications of key findings; systematic review registration number.	2
INTRODUCTION			
Rationale	3	Describe the rationale for the review in the context of what is already known.	3
Objectives	4	Provide an explicit statement of questions being addressed with reference to participants, interventions, comparisons, outcomes, and study design (PICOS).	3
METHODS			
Protocol and registration	5	Indicate if a review protocol exists, if and where it can be accessed (e.g., Web address), and, if available, provide registration information including registration number.	N/A
Eligibility criteria	6	Specify study characteristics (e.g., PICOS, length of follow-up) and report characteristics (e.g., years considered, language, publication status) used as criteria for eligibility, giving rationale.	3
Information sources	7	Describe all information sources (e.g., databases with dates of coverage, contact with study authors to identify additional studies) in the search and date last searched.	3
Search	8	Present full electronic search strategy for at least one database, including any limits used, such that it could be repeated.	3
Study selection	9	State the process for selecting studies (i.e., screening, eligibility, included in systematic review, and, if applicable, included in the meta-analysis).	3
Data collection process	10	Describe method of data extraction from reports (e.g., piloted forms, independently, in duplicate) and any processes for obtaining and confirming data from investigators.	3
Data items	11	List and define all variables for which data were sought (e.g., PICOS, funding sources) and any assumptions and simplifications made.	3
Risk of bias in individual	12	Describe methods used for assessing risk of bias of individual studies (including specification of whether this was	4

studies		done at the study or outcome level), and how this information is to be used in any data synthesis.	
Summary measures	13	State the principal summary measures (e.g., risk ratio, difference in means).	4
Synthesis of results	14	Describe the methods of handling data and combining results of studies, if done, including measures of consistency (e.g., I^2) for each meta-analysis.	4
Risk of bias across studies	15	Specify any assessment of risk of bias that may affect the cumulative evidence (e.g., publication bias, selective reporting within studies).	4
Additional analyses	16	Describe methods of additional analyses (e.g., sensitivity or subgroup analyses, meta-regression), if done, indicating which were pre-specified.	4
RESULTS			
Study selection	17	Give numbers of studies screened, assessed for eligibility, and included in the review, with reasons for exclusions at each stage, ideally with a flow diagram.	4
Study characteristics	18	For each study, present characteristics for which data were extracted (e.g., study size, PICOS, follow-up period) and provide the citations.	4
Risk of bias within studies	19	Present data on risk of bias of each study and, if available, any outcome level assessment (see item 12).	5
Results of individual studies	20	For all outcomes considered (benefits or harms), present, for each study: (a) simple summary data for each intervention group (b) effect estimates and confidence intervals, ideally with a forest plot.	5
Synthesis of results	21	Present results of each meta-analysis done, including confidence intervals and measures of consistency.	5
Risk of bias across studies	22	Present results of any assessment of risk of bias across studies (see Item 15).	5
Additional analysis	23	Give results of additional analyses, if done (e.g., sensitivity or subgroup analyses, meta-regression [see Item 16]).	5
DISCUSSION			
Summary of evidence	24	Summarize the main findings including the strength of evidence for each main outcome; consider their relevance to key groups (e.g., healthcare providers, users, and policy makers).	6
Limitations	25	Discuss limitations at study and outcome level (e.g., risk of bias), and at review-level (e.g., incomplete retrieval of identified research, reporting bias).	6
Conclusions	26	Provide a general interpretation of the results in the context of other evidence, and implications for future research.	7
FUNDING			
Funding	27	Describe sources of funding for the systematic review and other support (e.g., supply of data); role of funders for the systematic review.	1

Table S2. Search strategy in MEDLINE via Pubmed.

(covid or COVID-19 OR coronavirus OR "corona virus" OR SARSCoV-2 OR "Coronavirus"[Mesh] OR "severe acute respiratory syndrome coronavirus 2"[Supplementary Concept] OR "COVID-19"[Supplementary Concept] OR "Coronavirus Infections/epidemiology"[Mesh] OR "Coronavirus Infections/prevention and control"[Mesh] OR "Coronavirus Infections/psychology"[Mesh] OR "Coronavirus Infections/statistics and numerical data"[Mesh]) AND (anxiety OR anxiety symptoms OR anxiety disorders OR anxious OR "Trauma and Stressor Related Disorders"[Mesh] OR "Anxiety"[Mesh] OR "Anxiety Disorders"[Mesh] OR "Anxiety/epidemiology"[Mesh] OR "Anxiety/statistics and numerical data"[Mesh] OR depression OR depressive OR "Depression"[Mesh] OR "Depressive Disorder"[Mesh] OR "Depression/statistics and numerical data"[Mesh]) AND (“healthcare workers” OR “medical staff” OR “healthcare professionals” OR “health care workers” OR “health workers” OR “health professionals” OR “health personnel” OR "Health Personnel"[Mesh])

Table S3. Quality assessment with the JBI Appraisal Checklist for Prevalence Studies.

Study	1	2	3	4	5	6	7	8	9	TOTAL
Almater et al. (2020)	Y	N	N	Y	Y	Y	Y	Y	N	6
An et al. (2020)	Y	N	Y	Y	Y	Y	Y	Y	N	7
Cai et al. (2020)	Y	N	Y	Y	Y	Y	Y	Y	N	7
Chen J. et al. (2020)	Y	N	Y	Y	Y	Y	Y	Y	N	7
Chen Y. et al. (2020)	Y	U	N	Y	Y	Y	Y	Y	Y	7
Chew et al. (2020)	Y	U	Y	Y	Y	Y	Y	Y	Y	8
Dal'Bosco et al. (2020)	Y	N	N	Y	Y	Y	Y	Y	N	6
Di Tella et al. (2020)	Y	N	N	Y	Y	Y	Y	Y	N	6
Dosil Santamaría et al. (2020)	Y	N	Y	Y	Y	Y	Y	Y	N	7
Elbay et al.(2020)	Y	N	Y	Y	Y	Y	Y	Y	N	7
Elhadi et al. (2020)	Y	N	Y	Y	Y	Y	Y	Y	Y	8
Gallopeni et al. (2020)	Y	U	Y	Y	Y	Y	Y	Y	N	7
Gupta A.K. et al. (2020)	Y	N	N	Y	Y	Y	Y	Y	N	6
Gupta S. et al. (2020)	Y	N	Y	Y	Y	Y	Y	Y	Y	8
Huang & Zhao (2020)	Y	N	Y	N	Y	Y	Y	Y	Y	7
Kannampallil et al. (2020)	Y	U	Y	N	Y	Y	Y	Y	N	6
Keubo et al. (2020)	Y	N	N	Y	Y	Y	Y	Y	N	6
Khanna et al. (2020)	Y	U	Y	Y	Y	Y	Y	Y	N	7
Koksal et al. (2020)	Y	U	Y	Y	Y	Y	Y	Y	N	7
Krammer et al. (2020)	Y	N	N	Y	Y	Y	Y	Y	Y	7
Lai et al. (2020)	Y	N	Y	Y	Y	Y	Y	Y	Y	8
Lam et al. (2020)	Y	N	Y	N	Y	Y	Y	Y	Y	7
Li G. et al. (2020)	Y	N	Y	Y	Y	Y	Y	Y	Y	8
Liang et al. (2020)	Y	N	Y	Y	Y	Y	Y	Y	N	7
Lin et al. (2020)	Y	N	Y	N	Y	Y	Y	Y	N	6
Liu Y. et al. (2020)	Y	N	Y	Y	Y	Y	Y	Y	N	7
Lu et al. (2020)	Y	U	Y	Y	Y	Y	Y	Y	Y	8
Luceño-Moreno et al. (2020)	Y	N	Y	Y	Y	Y	Y	Y	Y	8
Magnavita et al. (2020)	Y	N	Y	Y	Y	Y	Y	Y	Y	8
Naser et al. (2020)	Y	N	Y	Y	Y	Y	Y	Y	N	7
Ning et al. (2020)	Y	N	Y	Y	Y	Y	Y	Y	N	7
Pouralizadeh et al. (2020)	Y	U	Y	Y	Y	Y	Y	Y	N	7
Que et al. (2020)	Y	N	Y	Y	Y	Y	Y	Y	N	7
Sahin et al. (2020)	Y	N	Y	Y	Y	Y	Y	Y	N	7
Salopek-Žiha et al. (2020)	Y	N	N	N	Y	Y	Y	Y	N	5
Sandesh et al. (2020)	Y	N	N	N	Y	Y	Y	Y	N	5
Si et al. (2020)	Y	N	Y	Y	Y	Y	Y	Y	Y	8
Song et al. (2020)	Y	N	Y	Y	Y	Y	Y	Y	N	7
Stojanov et al. (2020)	Y	U	N	Y	Y	Y	Y	Y	N	6
Suryavanshi et al. (2020)	Y	N	N	Y	Y	Y	Y	Y	N	6
Teng et al. (2020)	Y	N	Y	N	Y	Y	Y	Y	N	6
Teo et al. (2020)	Y	U	N	Y	Y	Y	Y	Y	Y	7
Tu et al. (2020)	Y	Y	N	Y	Y	Y	Y	Y	Y	8

**Vanni et al. (2020)**	Y	N	N	Y	Y	Y	Y	Y	Y	7
**Wang H. et al. (2020)**	Y	N	Y	N	Y	Y	Y	Y	Y	7
**Wang L.Q. et al. (2020)**	Y	N	N	Y	Y	Y	Y	Y	N	6
**Wang S. et al. (2020)**	Y	N	N	Y	Y	Y	Y	Y	Y	7
**Wang W. et al. (2020)**	Y	N	Y	Y	Y	Y	Y	Y	Y	8
**Wankowicz et al. (2020)**	Y	U	Y	Y	Y	Y	Y	Y	N	7
**Xiao et al. (2020)**	Y	N	Y	N	Y	Y	Y	Y	N	6
**Xiaoming et al. (2020)**	Y	N	Y	Y	Y	Y	Y	Y	Y	8
**Xiong et al. (2020)**	Y	N	N	Y	Y	Y	Y	Y	Y	7
**Yang S. et al. (2020)**	Y	N	N	Y	Y	Y	Y	Y	Y	7
**Zhang C. et al. (2020)**	Y	N	Y	Y	Y	Y	Y	Y	Y	8
**Zhou et al. (2020)**	Y	U	Y	Y	Y	Y	Y	Y	N	7
**Zhu J. et al. (2020)**	Y	N	N	Y	Y	Y	Y	Y	N	6
**Zhu Z. et al. (2020)**	Y	N	Y	Y	Y	Y	Y	Y	Y	8

Abbreviations: N: No; U: Unclear; Y: Yes; 1: Was the sample frame appropriate to address the target population?; 2: Were study participants recruited in an appropriate way?; 3: Was the sample size adequate?; 4: Were the study subjects and setting described in detail?; 5: Was data analysis conducted with sufficient coverage of the identified sample?; 6: Were valid methods used for the identification of the condition?; 7: Was the condition measured in a standard, reliable way for all participants?; 8: Was there appropriate statistical analysis?; 9: Was the response rate adequate, and if not, was the low response rate managed appropriately?
